# A Th1-like CD4^+^ T-cell Cluster That Predicts Disease-free Survival in Early-stage Lung Cancer

**DOI:** 10.1158/2767-9764.CRC-23-0167

**Published:** 2023-07-19

**Authors:** Akitoshi Yanagihara, Satoshi Yamasaki, Kosuke Hashimoto, Ryo Taguchi, Tetsuya Umesaki, Hisao Imai, Kyoichi Kaira, Hiroyuki Nitanda, Hirozo Sakaguchi, Hironori Ishida, Kunihiko Kobayashi, Katsuhisa Horimoto, Hiroshi Kagamu

**Affiliations:** 1 Department of General Thoracic Surgery, Saitama Medical University International Medical Center, Hidaka, Saitama, Japan.; 2 Department of Respiratory Medicine, Saitama Medical University International Medical Center, Hidaka, Saitama, Japan.; 3 Department of Clinical Cancer Genomics, Saitama Medical University International Medical Center, Hidaka, Saitama, Japan.; 4 Artificial Intelligence Research Center, National Institute of Advanced Industrial Science and Technology, Koto-ku, Tokyo, Japan.

## Abstract

Perioperative immune checkpoint inhibitors have been shown to improve prognosis in early-stage lung cancer. However, no biomarkers are known to indicate the requirement for treatment. This study aimed to identify T-cell clusters responsible for antitumor immunity in patients with early-stage lung cancer. Preoperative blood samples from 50 consecutive patients with lung cancer who were diagnosed as operable and underwent complete resection were analyzed by mass cytometry. Patients were divided into two groups: no recurrence at a minimum observation period of 851 days (median observation period: 1,031.5 days) and recurrence by the last observation date. Mass cytometry and single-cell RNA sequencing analysis of lymph nodes (LN) and tumor-infiltrating T cells were also performed. CCR4^−^CCR6^+^ Th7R showed discriminative ability between recurrence and non-recurrence patients with lung cancer. Patients with more than 3.04% Th7R showed significantly favorable disease-free survival. Th7R was a major component of CD4^+^ T cells in tumor microenvironments and LNs adjacent to lung cancer tissues and was the only cluster that decreased in peripheral blood after the removal of cancer tissues, suggesting that Th7R was primed and proliferated in tumor-draining LNs in the presence of cancer tissues. Th7R had the kinetics that antitumor T cells should have, as indicated by the cancer immunity cycle; thus, peripheral blood Th7R could represent the potency of tumor immunity by reflecting priming and proliferation in tumor-draining LNs and Th7R in the tumor microenvironment. Prediction using peripheral Th7R before surgery could allow the selection of patients who require perioperative drug therapy and optimize therapeutic interventions with clinical implications.

**Significance:**

Peripheral Th7R, a Th1-like CD4^+^ T-cell cluster reflecting priming status in draining LNs and immune status in the tumor microenvironment, predicts disease-free survival after complete resection and has significant clinical relevance in selecting appropriate therapeutic interventions in patients with early-stage lung cancer.

## Introduction

The cancer immunoediting theory suggests that immortalized cells resulting from the accumulation of genetic mutations are recognized by the immune system in the early stages of transformation. The power relationship between tumor cells and the immune system progresses over time from an elimination phase in which the immune system dominates, followed by an equilibrium phase, to an escape phase in which tumor cells dominate and allow tumor tissue formation (1). T-cell immunity has been shown to support the equilibrium phase, and the weakening of T-cell immunity is necessary for establishing tumor tissues.

Cancer progression begins with the formation of tumor tissue at the primary site, where immortalized cells arise, and progresses over time to metastasis to nearby lymph nodes (LN) and then to distant organs. Because lung cancer also progresses according to this principle, staging is based on information from the primary tumor, LN metastasis, and distant metastasis. Surgical resection is the standard treatment for lung cancer that is restricted to the primary tumor or progressed to ipsilateral hilar LNs. Therefore, compared with advanced stages, operable early-stage cancers are more likely to have T-cell immunity closer to the equilibrium phase. Perioperative immune checkpoint inhibitor (ICI) therapy that takes advantage of this has been reported to achieve high pathologic complete response rates (2, 3).

T-cell exhaustion is an important mechanism of T-cell immune weakening from the equilibrium phase to the escape phase, and immune checkpoint molecules such as programmed cell death-1 (PD-1) and CTL antigen 4 (CTLA-4), which are expressed in T cells and impair their function, have been shown to play a critical role in T-cell exhaustion. ICI therapy targeting checkpoint molecules, such as PD-1 and CTLA-4, has achieved unprecedented long-term survival benefits in advanced lung cancer (4–6). However, relatively few cases benefit, and it is believed that a prespecified T-cell immune status is required to achieve sufficient antitumor T-cell immune responses with ICI therapy. PD-1^+^ CD8^+^ T cells in tumor tissues are required for this T-cell immune status, and recently, transcription factor 7 (TCF7)-positive PD-1^+^ precursor-exhausted CD8^+^ T cells have been considered important for the therapeutic efficacy of PD-1 blockade (7, 8). In contrast, the presence of expanded T-cell clones in the peripheral blood, which are shared with T cells in the tumor microenvironment (TME) and associated with ICI response, has led to the belief that T cells are continuously recruited from secondary lymphoid organs to TME through the peripheral blood according to the cancer immune cycle even in the cancer host in the escape phase, and are responsible for antitumor T-cell immunity.

CD8^+^ T cells recognize MHC class I-restricted antigens and exert their cytotoxic effects. Their priming, clonal proliferation, peripheral blood migration, tumor infiltration, and cytotoxic activities are assisted by CD4^+^ T cells, with CD4^+^ T cells and CD8^+^ T cells mediated by normal type 1 dendritic cells (cDC1; refs. 9–11). CD4^+^ T cells undergo polarization into type 1 Th (Th1), Th2, and Th17 cells to accomplish multiple systemic tasks depending on the type of infectious microorganism being addressed and eliminate targets most efficiently (12, 13). Although Th1 has been considered important in antitumor immunity, viability, as represented by Th17, has also been considered important. Clinical studies have repeatedly reported the importance of Th1-like CD4^+^ T cells in antitumor immunity, but the differences with Th1 remain unclear. Using single-cell RNA sequencing (scRNA-seq) and mass cytometry analysis of peripheral blood from patients with advanced lung cancer treated with PD-1 blockade, we identified a unique T-bet–positive Th1-like cluster that differs from Th1 in the epigenome, T-cell receptor (TCR) clonotype, and gene expression pattern (14). The Th1-like cluster consisting of CXC motif chemokine receptor 3^+^ (CXCR3^+^), CC chemokine receptor 4^−^ (CCR4^−^) CCR6^+^ cells, and CXCR3^−^CCR4^−^CCR6^+^ cells was considered to be one functional meta-cluster, as it formed one distinct node in the trajectory analysis with Th1 and Th17 gene expression as both ends. We called the Th1-like cluster Th7R after the high expression of the IL7 receptor. Th7R but not Th1 in the peripheral blood before therapy correlated with progression-free survival and overall survival after PD-1 blockade treatment.

In this study, we analyzed tumor-associated LNs, peripheral blood, and tumor-infiltrating lymphocytes (TIL) from patients with early-stage lung cancer surgery, whose immune status is considered to be closer to the equilibrium phase, and examined the association between T-cell cluster dynamics, including Th7R, and postoperative disease-free survival (DFS).

## Materials and Methods

### Patients and Treatment

To analyze clinical outcomes according to T-cell immune cluster status, peripheral blood was collected from 50 consecutive patients with non–small cell lung cancer who underwent radical surgery between October 2017 and October 2019 at Saitama Medical University International Medical Center. These patients comprised the “clinical analysis cohort.” LN samples were also collected from 20 of this cohort's patients in whom LN metastasis had not occurred. Surgery was performed by way of lobectomy, regional LN dissection, and R2 LN sampling. TILs and peripheral blood were collected from 4 patients with stage Ⅰ non–small cell lung cancer; these patients comprised the “tissue cohort” (2 male and 2 female patients; age: 65–80 years). Patient demographics are shown in Table 1.

**TABLE 1:** Patient characteristics

	Tissue cohort (*n* = 4)	Clinical analysis cohort (*n* = 50)
Age, years		
Median	72	71
Range	65–80	50–83
Sex, *n* (%)		
Male	2 (50.0)	31 (62.0)
Female	2 (50.0)	19 (38.0)
p-stage, *n* (%)		
I	4 (100)	42 (84.0)
II	0 (0)	3 (6.0)
III	0 (0)	5 (10.0)
Smoking history, *n* (%)		
Current or former smoker	2 (50)	32 (64.0)
Never smoked	2 (50)	18 (36.0)
Histology, *n* (%)		
Sq	1 (25.0)	6 (12.0)
Non-Sq	3 (75.0)	44 (88.0)
PD-L1 expression level, *n* (%)		
<1%	0 (0)	12 (24.0)
1%–49%	3 (75.0)	20 (40.0)
>50%	1 (25.0)	8 (16.0)
Unknown	0 (0)	10 (20.0)
EGFR mutation, *n* (%)		
Yes	0 (0)	25 (50.0)
No	4 (100)	25 (50.0)

The patients were evaluated for recurrence using a CT scan every 4–6 months, with a final observation date of January 31, 2022. During this period, 13 patients had lung cancer recurrence. All specimens were collected after obtaining written informed consent. The Internal Review Board of Saitama Medical University International Medical Center approved the study protocol in accordance with the Declaration of Helsinki (approval no.: 17-084).

### Blood Sample Analysis

Peripheral blood samples were collected before and a week after surgery, using heparinized CPT Vacutainer tubes (Becton Dickinson Vacutainer Systems), as described previously (15). The samples were frozen using Cellbanker2 (Nippon Zenyaku Kogyo Co.) in a liquid nitrogen tank. For analyses of T-cell subsets, cells were incubated for 32–48 hours in culture medium consisting of RPMI1640 medium supplemented with 10% FCS before cell staining.

### LN Collection

Station #12 hilar LNs nearest to the lung cancer tissues were collected as draining LNs. The ipsilateral mediastinal LNs were collected as distant LNs. Single-cell suspensions were prepared mechanically by teasing the collected LNs with needles and pressing tissue fragments with the blunt end of a 10-mL plastic syringe.

### TILs

TILs were prepared as follows. The collected tumors were cut into 2–3 mm pieces and incubated with DMEM supplemented with Dri Tissue & Tumor Dissociation Reagent (BD horizon: 661563, BD Biosciences) or with RPMI1640 medium supplemented with Liberase TL Research Grade (05401020001, Roche), according to the manufacturer's instructions. Biotin anti-human CD235ab antibody and streptavidin nanobeads (480016, BioLegend) were used to remove erythrocytes, according to the manufacturer's instructions.

### Mass Cytometry

The mAbs used for Helios mass cytometry analysis are listed in Supplementary Table S1. Cell preparation and measurement with Helios were performed according to the manufacturer's instructions (Standard BioTools). Briefly, up to 5.0 × 10^6^ cells were stained with mass cytometry antibodies. For intracellular staining, samples were prepared using a Maxpar Nuclear Antigen Staining Buffer before staining. After washing twice with Maxpar Cell Staining Buffer, samples were fixed with Maxpar Fix and Perm Buffer supplemented with a 125-nmol/L iridium nucleic acid intercalator (Standard BioTools). Following fixation, the cells were washed once with Maxpar Cell Staining Buffer and twice with Maxpar water and then resuspended in Maxpar water. More than 200,000 cells per sample were analyzed using Helios and Cytobank (https://www.cytobank.org) software. The gating strategy is presented in Supplementary Fig. S1.

### scRNA-seq

All scRNA-seq libraries were constructed using the Chromium Single Cell Immune Profiling v2 kit (10× Genomics Inc.) All libraries were sequenced on DNBSEQ-G400 sequencers (MGI) as paired-end mode (read1: 28 bp; read2: 90 bp). All library construction and sequencing were performed using the manufacturer's protocol.

The sequenced reads were processed using Cell Ranger 6 software and the GRCh38 reference dataset (version 2020-A for gene expression and 5.0.0 for the TCR repertoire). Quality control, statistical analysis, and graphical drawing were conducted using the Loupe browser (10× Genomics Inc.) and the Seurat 4.0 package in R (4.0.3; https://satijalab.org/seurat/; R Foundation for Statistical Computing; refs. 16, 17), in the same method as described previously (14). In this study, cells with a valid TCR clonotype in the CD3D^+^ cluster were selected using Loupe browser in the first step. Data integration and unsupervised clustering of all derived CD3^+^ T cells were performed using Seurat standard workflow in the same method as described previously (14). The differential expression analysis between Th1 type cluster and Th7R type cluster, which were annotated according to the expression pattern of several signature surface proteins, were performed using the MAST package in R software (18), which is implemented in the FindMarkers function of the Seurat package.

### Statistical Analysis

Prism 9 (GraphPad Software) was used to conduct statistical analyses. Data are expressed as the mean ± SEM unless otherwise indicated. Tests for differences between the two populations were performed using Welch *t* test. Multiple group comparisons were conducted using one-way ANOVA with Tukey *post hoc* analysis. Survival curves were estimated using the Kaplan–Meier method. Tests for differences and HR were conducted using the log-rank (Mantel–Cox) test. All *P* values were two sided, and *P* < 0.05 was considered significant, except for the numerical correlation of T-cell clusters between peripheral blood and TILs. For the T-cell cluster numerical linear correlation test, *P* < 0.1 and *r* > 0.9 were considered significant.

### Data Availability

The RAW data of scRNA-seq generated in this study are publicly available in Gene Expression Omnibus at GSE215219.

## Results

### Peripheral CD62L^low^ Th7R Subpopulation Predicts DFS in Patients with Early-stage Lung Cancer After Surgery

**FIGURE 1:**
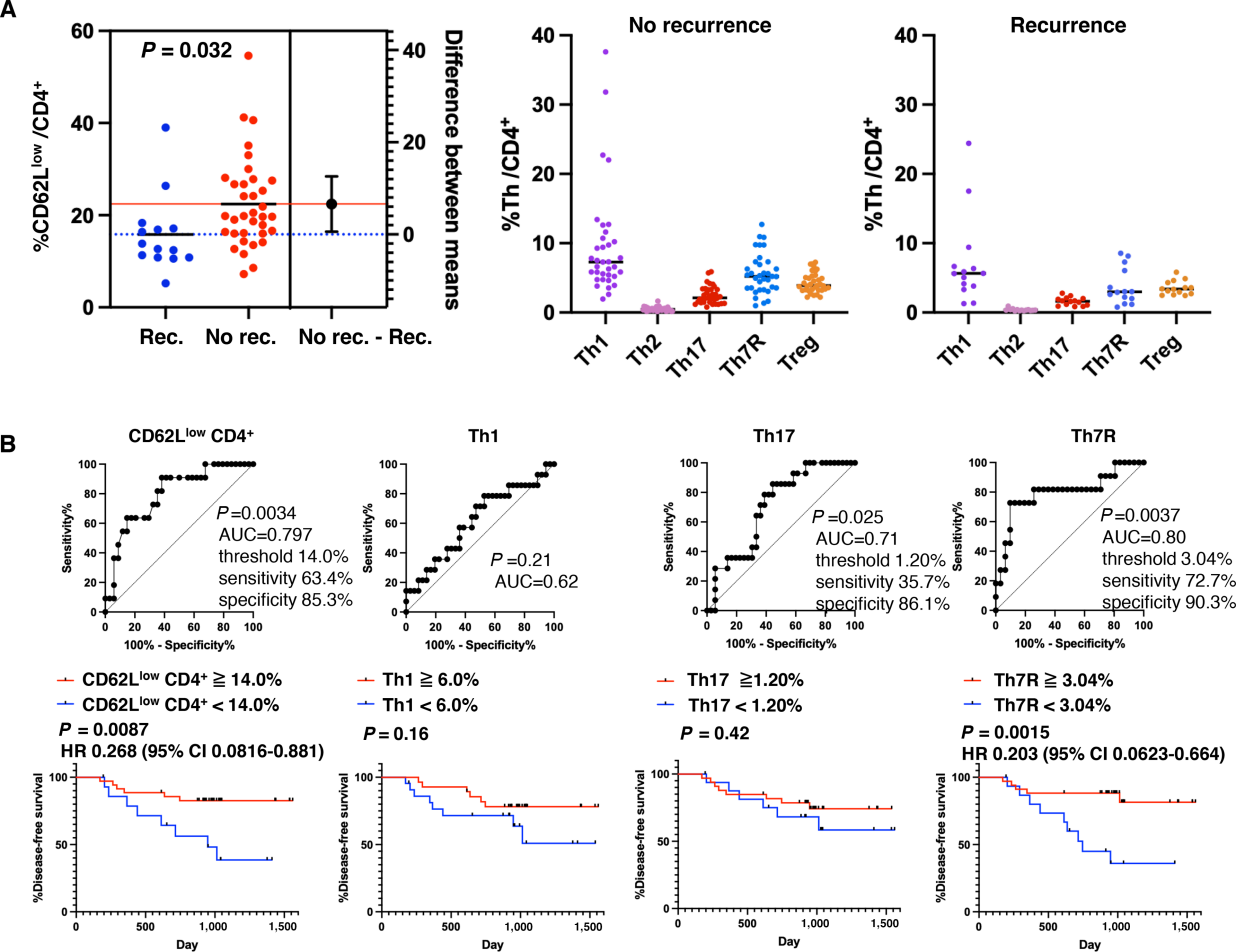
CD4^+^ T-cell clusters predict DFS of patients with lung cancer after surgical resection. **A,** Peripheral blood T cells collected from patients with lung cancer before surgery were analyzed by mass cytometry. The percentage of CD62L^low^ CD4^+^ T cells and each Th cell type in the CD62L^low^ CD4^+^ T-cell subpopulation is shown for patients who relapsed by the last observation date and for those who did not relapse. Th types were determined by chemokine expression patterns, including CXCR3, CCR4, and CCR6. Unpaired Welch *t* test was used for statistical analysis. **B,** ROC curve analysis was performed to determine whether Th clusters in the CD62L^low^ CD4^+^ T-cell subpopulation could discriminate between relapsed and non-relapsed patients. Next, Kaplan–Meier analysis of DFS was performed using the threshold obtained from ROC analysis as the best likelihood ratio, or the median value was used as a threshold if ROC analysis showed no significance. Significance was tested by a log-rank (Mantel–Cox) test. The HR and 95% CI were obtained by the log-rank test.

Preoperative blood samples from 50 consecutive patients with lung cancer who were diagnosed as operable and underwent complete resection between October 2017 and October 2019 at Saitama Medical University International Medical Center were analyzed by mass cytometry. Patients were divided into two groups, one with no recurrence at a minimum observation period of 851 days (median observation period: 1,031.5 days) and the other with recurrence by the last observation date, and the results of T-cell clusters were compared (Table 1; Fig. 1A). The percentage of effector CD4^+^ T cells that downregulated CD62 L expression (%CD62L^low^/CD4^+^) was significantly higher in the recurrence-free group (*P* = 0.032). The percentage of Th cells comprising the CD62L^low^CD4^+^ T-cell subpopulation was annotated by the expression patterns of CXCR3, CCR4, and CCR6. The CXCR3^+^CCR4^−^CCR6^−^ Th1 type was the most common in both the recurrence-free and recurrence groups, followed by the CXCR3^±^CCR4^−^CCR6^+^ Th7R type. In a previous study, the Th7R cluster, composed of CXCR3^+^CCR4^−^CCR6^+^ Th1/17 and CXCR3^−^CCR4^−^CCR6^+^ CCR6 single positive (CCR6 SP), was identified as a Th1-like CD4^+^ T-cell cluster predictive of the efficacy of PD-1 blockade therapy in advanced lung cancer, characterized by expression of IL7 receptor, TCF7, and granzyme K (GZMK), and distinct from Th1 in gene expression, epigenetics, and TCR clonotype (14). Th1 and Th7R were followed by the CXCR3^−^CCR4^+^CCR6^+^ Th17 type and a small number of the CXCR3^−^CCR4^+^CCR6^−^ Th2 type. This Th cell percentage pattern was consistent with the results of peripheral blood analysis of advanced-stage lung cancer cases.

ROC curve analysis was performed to determine the discriminative ability of T-cell clusters between recurrence and non-recurrence groups (Fig. 1B). The percentage of CD62L^low^ CD4^+^ T cells showed significant discriminatory power (*P* = 0.0034) with a sensitivity of 63.4% and specificity of 85.3% at a threshold of 14.0%. Kaplan–Meier analysis of DFS using this threshold showed a significant HR of 0.268 [95% confidence interval (CI), 0.0816–0.881] in the log-rank test with better DFS in the %CD62L^low^/CD4^+^ ≥ 14.0% group. Next, the percentage of Th cells comprising the CD62L^low^ CD4^+^ T-cell subpopulation was included in the analysis. Th1 did not show significant discriminatory power in the ROC analysis, and the Kaplan–Meier analysis using median values did not show significant differences in DFS. Th17 showed significant discriminatory ability in the ROC analysis (*P* = 0.025) but did not show a significant difference in DFS in the Kaplan–Meier analysis using the threshold with the highest likelihood ratio. In contrast, Th7R showed significant discrimination (*P* = 0.0037) between recurrence and non-recurrence with a sensitivity of 72.7% and specificity of 90.3% at a threshold of 3.04%. Kaplan–Meier analysis showed significantly (*P* = 0.0015) better DFS in the %Th7R ≥ 3.04% group than the %Th7R < 3.04% group, with an HR of 0.203 (95% CI, 0.0623–0.664). Subgroup analysis showed that the HR was even better in stage Ⅰ–Ⅱ patients (*P* < 0.0001; HR, 0.1003; 95% CI, 0.0236–0.427; Supplementary Fig. S2A), and that the discriminatory ability was effective for both EGFR wild-type lung cancer and lung cancer harboring activating EGFR mutations (Supplementary Fig. S2B).

For CD8^+^ T cells, the same analysis was performed by dividing them into four clusters according to CD62 L and CD45RA expression: CD62L^high^CD45RA^+^ naïve, CD62L^high^CD45RA^−^ central memory, CD62L^low^CD45RA^−^ effector memory (EM), and CD62L^low^CD45RA^+^ effector memory with CD45RA expression (EMRA; Supplementary Fig. S3A and S3B). ROC analysis showed no significant discriminative power. Kaplan–Meier analysis performed on median values showed a trend toward better DFS in the group with the lower percentage of naïve cells and the group with the higher percentage of EMRA cells, but the difference was not significant

### CD62L^low^ Th1 and Th7R Comprise CD4^+^ T Cells in the TME

**FIGURE 2:**
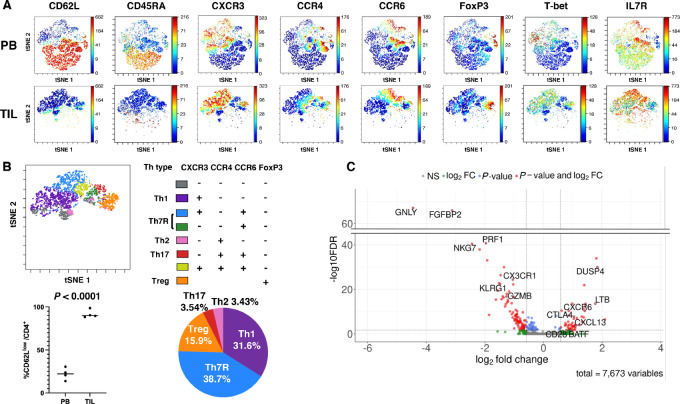
CD4^+^ T-cell clusters in TILs. **A,** TIL T cells were analyzed by mass cytometry. TIL and preoperative peripheral blood T-cell data were compiled, processed by clustering into 10 clusters, and then annotated on the basis of CXCR3, CCR4, CCR6, and FoxP3 expression. **B,** Representative annotation of TIL-tSNE using CXCR3, CCR4, CCR6, and FoxP3, comparison of the percentage of CD62L^low^ CD4^+^ T cells between peripheral blood and TIL, and pie chart showing the average percentage of TIL-CD4^+^ T-cell clusters are shown. **C,** Volcano plot of the differential expression analysis between the TIL-Th7R and TIL-Th1 clusters derived from scRNA-seq analysis. Genes with FDR (adjusted *P* value) < 0.02 and fold change > 1.5 are represented by red dots. Genes with positive log_2_ fold change were highly expressed in TIL-Th7R.

It is known that the postoperative prognosis is better when there are more lymphocytes infiltrating the lung cancer tissue (19). Therefore, to clarify the relationship between peripheral blood T-cell clusters, which could discriminate postoperative recurrence-free cases and TIL clusters, we performed t-distributed stochastic neighbor embedding (tSNE) analysis of peripheral blood and TILs simultaneously for comparative analysis with the tissue cohort (*n* = 4) using mass cytometry data (Fig. 2A). Although CD62L^high^ and CD62L^low^ subpopulations were found in the peripheral blood, TILs were predominantly composed of the CD62L^low^ subpopulation. In addition, FlowSOM annotation showed that TIL-CD4^+^ T cells were broadly classified into FoxP3^+^ regulatory T cells (Treg) and the effector fraction and the effector fraction consisted mainly of Th1 and Th7R (Fig. 2B).

To clarify the distinct roles of Th1 and Th7R in the TME, we next analyzed gene expression profiles by scRNA-seq. Th1-dominant expression was observed in genes involved in cytotoxic activity, such as *GZMB*, *PRF1*, *NKG7*, and *GNLY*, while Th7R expressed *CXCL13* and *LTB*, which are thought to be involved in the formation of high endothelial venules (HEV) and tertiary lymphoid structures (TLS; refs. 20, 21). *CTLA-4*, *CD28*, *BATF*, *DUSP4,* and *CXCR6* were also significantly upregulated in Th7R predominance (Fig. 2C; Supplementary Table S2).

**FIGURE 3:**
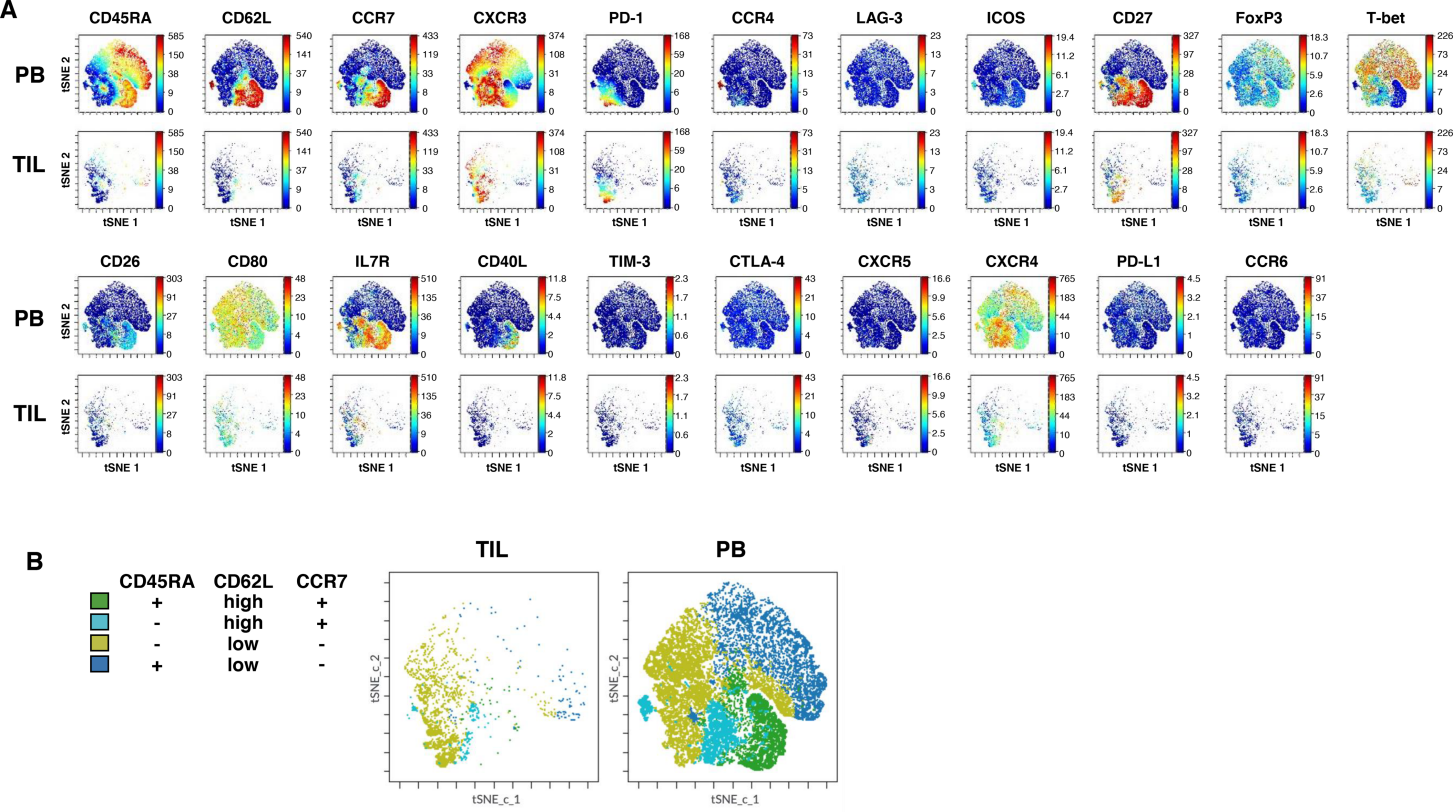
CD8^+^ T-cell clusters in TILs. **A,** Representative cell-level molecular expression using tSNE figures by mass cytometry analysis of gated CD8^+^CD3^+^ TILs and peripheral blood cells. **B,** FlowSOM annotation using CD45RA, CD62L, and CCR7 expression is shown.

Most CD8^+^ T cells in TILs showed an EM phenotype with CD62L^low^, CCR7^−^, and CD45RA^−^ (Fig. 3A and B). In addition, a few CD45RA^−^ CD8^+^ T-cell clusters with PD-1, CD62L, and CCR7 expression were observed.

### Th7R is a Major Component of Primed CD4^+^ T Cells in Lung Cancer Draining LNs

**FIGURE 4:**
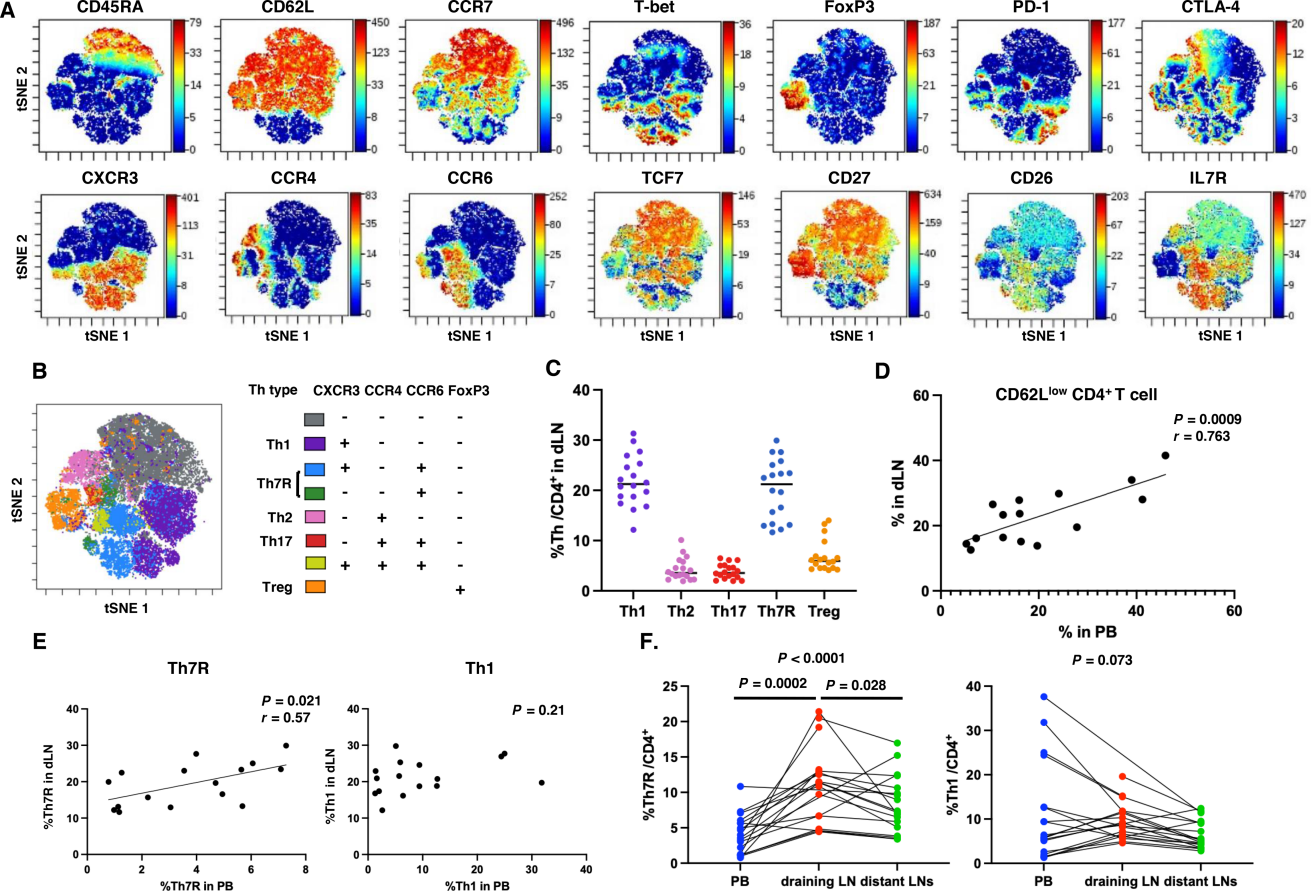
CD4^+^ T-cell clusters in LNs. **A,** Cells from station #12 LNs close to lung cancer tissue were analyzed by mass cytometry as draining LNs, and cells from ipsilateral mediastinal LNs were analyzed as distant LNs (*n* = 20). Representative tSNE figures of gated CD4^+^CD3^+^ cells of lung cancer draining lymph node cells. **B,** Representative annotation of draining LN cells according to CXCR3, CCR4, CCR6, and FoxP3 expression. **C,** The percentages of Th and Treg clusters in draining LNs (dLN). **D,** CD62L^low^ CD4^+^ T-cell correlation between peripheral blood (PB) and draining LNs was analyzed. **E,** Correlation between the percentage of CD62L^low^ Th in PB and that of total Th in draining LNs was analyzed. **F,** The percentages of Th7R and Th1 between PB, draining LNs, and distant LNs were compared. Differences were tested using the mixed effects model (REML) followed by Tukey multiple comparison test.

According to the cancer immune cycle theory, cancer antigen-specific T-cell priming and clonal proliferation are thought to occur in secondary lymphoid organs where cancer-derived dendritic cells reach. Therefore, we analyzed the composition of T-cell clusters in station #12 LNs, which were closest to the cancer tissue, as draining LNs and mediastinal LNs as distant LNs in patients without LN metastasis (*n* = 20; Fig. 4A). Annotation results using CXCR3, CCR4, CCR6, and FoxP3 showed that Th1 and Th7R were the major components of the CD4^+^ T cells in draining LNs (Fig. 4B and C). The percentage of CD62L^low^CD4^+^ T cells showed a significant linear correlation in draining LNs and peripheral blood, consistent with the cancer immune cycle theory that effector T cells proliferating in LNs migrate to peripheral blood (Fig. 4D). The percentage of Th7R in peripheral blood was significantly correlated with total Th7R in draining LNs, but there was no correlation between draining LNs and peripheral blood in Th1 (Fig. 4E). Comparative analysis of the percentage of Th7R and Th1 cells in peripheral blood, draining LNs, and distant LNs revealed a significantly higher percentage of Th7R in draining LNs and no significant difference in Th1 (Fig. 4F), suggesting that Th7R cells were primed and proliferated in draining LNs.

**FIGURE 5:**
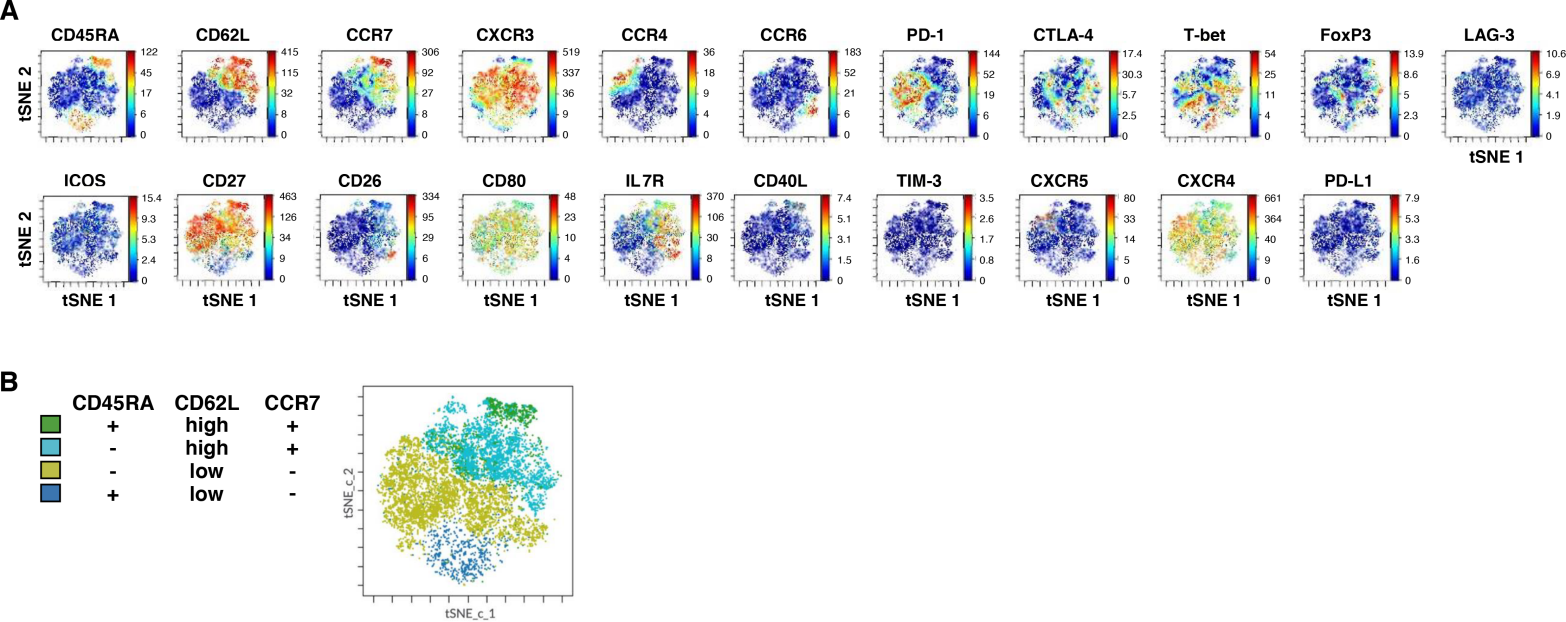
CD8^+^ T-cell clusters in LNs. **A,** Representative cell-level molecular expression using tSNE figures by mass cytometry analysis of gated CD8^+^CD3^+^ draining LNs. **B,** FlowSOM annotation using CD45RA, CD62L, and CCR7 expression is shown.

Draining LN CD8^+^ T cells contained few naïve cells, the majority being EM type, with the remainder being CD62L^high^CCR7^+^CD45RA^−^ and CD62L^low^CCR7^−^CD45RA^+^ cells (Fig. 5).

### CD62L^low^ Th7R Decreased After Complete Resection of Lung Cancer

**FIGURE 6:**
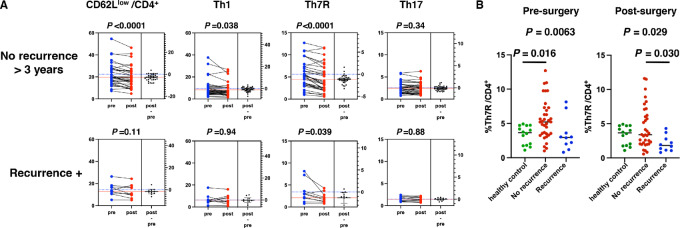
T-cell cluster changes after surgical resection. **A,** The percentages of CD62L^low^ CD4^+^ T cells and Th clusters in the CD62L^low^ CD4^+^ T-cell subpopulation were compared before and after surgical resection in patients with no recurrence and those with recurrence. Differences were tested using the paired Welch *t* test. **B,** The percentages of Th7R were compared between patients with no recurrence, patients with recurrence, and healthy controls. Differences were tested using ANOVA followed by Tukey multiple comparison test.

If Th7R cells are driven by cancer-derived antigens and proliferate in the regional LNs and migrate through the peripheral blood to infiltrate into the tumor, their proportion would be reduced in the peripheral blood by complete resection of lung cancer tissue and draining LNs. Therefore, we analyzed T-cell percentages in peripheral blood before and after surgery. Among CD8^+^ T cells, there was a significant postoperative increase in the naïve T-cell subpopulation in recurrence-free patients (*P* = 0.011) and a decrease in the CD62L^high^CD45RA^−^ cell subpopulation (*P* = 0.026). However, there were no significant changes in the proportions of CD62L^low^CD45RA^−^ and CD62L^low^CD45RA^+^ cells, referred to as effector memory or EMRA (Supplementary Fig. S4). In contrast, CD4^+^ T cells showed a significant decrease (*P* < 0.0001) in the percentage of CD62L^low^ CD4^+^ T cells after surgery in the recurrence-free group (Fig. 6A). Although Th1 cell percentage significantly decreased among the CD62L^low^ CD4^+^ T-cell subpopulation (*P* = 0.038), the greatest decrease was in the percentage of Th7R cells (*P* < 0.0001). The percentage of Th7R also significantly decreased in the recurrence group (*P* = 0.039). Next, the percentage of Th7R cells in the peripheral blood was compared preoperatively and postoperatively among the recurrence-free and recurrence groups and healthy controls (Fig. 6B). The preoperative percentage of Th7R in the recurrence-free patients was significantly higher than that in healthy controls (*P* = 0.016), but after surgery, there was no significant difference in the percentage between the groups. The recurrence patients had Th7R cell percentages similar to those of healthy subjects before surgery, and even after surgery, they had significantly lower Th7R cell percentages than the recurrence-free patients (*P* = 0.030).

## Discussion

The percentage of Th7R in peripheral blood, which correlates with response to PD-1 blockade therapy in advanced lung cancer study (14), predicted postoperative DFS in early-stage lung cancer in this study. These results suggest that postoperative recurrence is highly dependent on the patient's preoperative T-cell immune status, which can be assessed by peripheral Th7R. Th7R is a major component of CD4^+^ T cells in TILs and tumor-draining LNs. Particularly, more Th7R was observed in LNs close to the tumor rather than in LNs at distant sites, and the percentage of Th7R in draining LNs correlated with those in peripheral blood. This suggests that Th7R is primed in tumor-draining LNs and proliferate and migrate to the peripheral blood to infiltrate tumor tissue; thus, Th7R is thought to possess the kinetics that antitumor T cells should have, according to the cancer immunity cycle theory. In other words, peripheral blood Th7R may reflect priming and proliferation in tumor-draining LNs and tumor microenvironmental immune status.

The cancer immunoediting theory proposes that T-cell immunity, which controls cancer cells as few cells in the equilibrium phase, weakens, leading to an escape phase that allows tumor tissue formation. In advanced lung cancer, PD-1 blockade therapy and anti-CTLA-4 antibody drug therapy have resulted in long-term survival in 20%–30% of patients, and it is believed that the main mechanism by which T-cell immunity is weakened is T-cell exhaustion (6, 22). However, the current study suggests that one of the causes of weakened antitumor immunity in early-stage lung cancer is a decrease in Th7R numbers, and that the presence of sufficient Th7R suppresses postoperative recurrence. On the other hand, while Th7R was highly effective in predicting recurrence-free disease in stage I–II patients, stage III patients with confirmed mediastinal LN metastasis had recurrence even when sufficient numbers of Th7R were present. This suggests that, as in advanced-stage lung cancer, T-cell exhaustion is more involved in the decline of antitumor T-cell immunity in stage III patients. This is consistent with the fact that perioperative ICI therapy is more effective in preventing recurrence in stage III patients than in stage I–II patients (2, 3, 23).

Recently, perioperative drug therapy, with an EGFR-tyrosine kinase inhibitor (EGFR-TKI) such as osimertinib, has been developed (24). However, perioperative drug therapy is unnecessary for patients who are cured by surgery alone and only creates a risk of adverse events and an economic burden. The current study demonstrated the predictive ability of Th7R for recurrence in both EGFR wild-type and EGFR mutation-positive lung cancer. Thus, Th7R-mediated prediction of postoperative recurrence suggests the possibility of optimizing perioperative treatment with not only ICIs but also EGFR-TKIs.

We found that classical Th1 and Th7R were equally present in lung cancer tissues as Th1-like CD4^+^ T cells, with few Th2 and Th17 types. The classical Th1 expressed genes involved in cytotoxicity, such as *GZMB*, *PRF1*, *GNLY*, and *NKG7*, whereas Th7R expressed *CXCL13* and *LTB*, which are thought to be involved in HEV and TLS formation (20, 21, 25). Recently, HEV and TLS formation in TME is thought to play an important role in antitumor immunity. In secondary lymphoid organs, CD4^+^ non-T cells capable of producing CXCL13 and LTβ play a critical role in HEV formation (20). In contrast, it was reported that infiltration of CXCL13-producing CD4^+^ T cells precedes HEV and TLS formation in the ovarian cancer microenvironment (21). In this study, Th7R was the only cluster in which we detected significant CXCL13 gene expression in all CD4^+^ T-cell clusters in the lung cancer microenvironment. Recent studies of TILs using scRNA-seq have elucidated that effector CD8^+^ T-cell clusters with PD-1 and TCF7 expression, precursor exhausted CD8^+^ T cells (Tpex), play a critical role in antitumor immunity (7, 8). Intratumoral HEVs are thought to be important as a gateway for Tpex, which reexpress CD62 L and CCR7, to enter the TME (25). Taken together, Th7R is thought to mediate antitumor immunity not by direct cytotoxicity, but by recruiting antitumor CD8^+^ T cells such as Tpex through its involvement in HEV and TLS formation.

A limitation of this study is that it is an observational study, and we could not validate the direct antitumor effect of Th7R transfer. Whether Th7R is involved in HEV and TLS formation and whether antitumor effects are caused because of the transfer of cultivated Th7R need to be addressed in the future.

The prediction of recurrence-free survival in patients with early-stage lung cancer using Th7R may allow optimization of perioperative drug therapy and have clinical implications. In addition, this study showed that an insufficient number of Th7R resulted in the risk of recurrence after surgery. Thus, supplementation of Th7R as cell therapy for patients with early-stage lung cancer might improve the probability of recurrence-free survival.

## Supplementary Material

Supplementary Figure S1 Fig. S1. Gating strategy of CyTOF analysis

Supplementary Figure S2 Fig. S2. Kaplan-Meier analysis of disease-free survival (DFS) in patients with stage Ⅰ-Ⅱ disease (A), epidermal growth factor receptor (EGFR) wild-type disease, and EGFR mutations (B) using the threshold of preoperative %Th7R in peripheral blood obtained by receiver operating characteristic curve analysis. Significance was tested using the log-rank (Mantel-Cox) test. Hazard ratio (HR) and 95% confidence interval (CI) were obtained by log-rank test.

Supplementary Figure S3 Fig. S3. A: CD8+ T cells in peripheral blood collected before surgery were divided into four clusters according to CD62L and CD45RA expression and compared between no recurrence patients and recurrent patients: CD62LhighCD45RA+ naive, CD62LhighCD45RA- central memory (CM), CD62LlowCD45RA- effector memory (EM), and CD62LlowCD45RA+ effector memory with CD45RA expression (EMRA). B: Receiver operating characteristic curve (ROC) analysis was performed to determine if CD8+ T-cell clusters had the ability to discriminate between relapsed and non-relapsed patients. Then, Kaplan-Meier analysis of disease-free survival (DFS) was performed using the median value was used as a threshold because ROC analysis showed no significance. Significance was tested by log-rank (Mantel-Cox) test. Hazard ratio (HR) and 95% confidence interval (CI) were obtained by log-rank test.

Supplementary Figure S4 Fig. S4. The percentages of CD8+ T-cell clusters in the peripheral blood were compared before and after surgical resection in patients with no recurrence and patients with recurrence. Differences were tested by paired Welch's t-test

Table S1, Table S2 Table S1. List of antibodies used for CyTOF.
Table S2. Differentially expressed genes between Th7R cluster and Th1 cluster in tumor-infiltrating lymphocytes.
