# Supplementary figures and images for: A Th1-like CD4+ T-cell Cluster That Predicts Disease-free Survival in Early-stage Lung Cancer
Source: Cancer Res Commun. 2023 Jul 19;3(7):1277–85. doi: 10.1158/2767-9764.CRC-23-0167 (PMC10355164; doi:10.1158/2767-9764.CRC-23-0167)

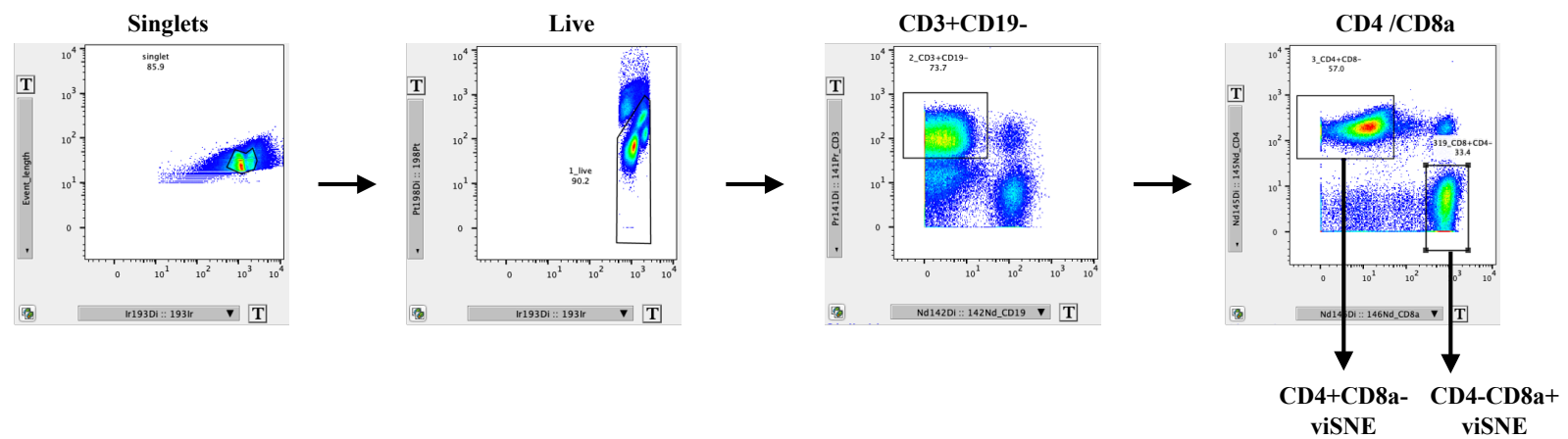

**Fig. S1.**  
Gating strategy of CyTOF analysis.

Supplement: Supplementary Figure S1 — Fig. S1. Gating strategy of CyTOF analysis [file crc-23-0167-s01.pdf]
