# Supplementary material for: A Th1-like CD4+ T-cell Cluster That Predicts Disease-free Survival in Early-stage Lung Cancer
Source: Cancer Res Commun. 2023 Jul 19;3(7):1277–85. doi: 10.1158/2767-9764.CRC-23-0167 (PMC10355164; doi:10.1158/2767-9764.CRC-23-0167)
Supplement: Supplementary Figure S2 — Fig. S2. Kaplan-Meier analysis of disease-free survival (DFS) in patients with stage Ⅰ-Ⅱ disease (A), epidermal growth factor receptor (EGFR) wild-type disease, and EGFR mutations (B) using the threshold of preoperative %Th7R in peripheral blood obtained by receiver operating characteristic curve analysis. Significance was tested using the log-rank (Mantel-Cox) test. Hazard ratio (HR) and 95% confidence interval (CI) were obtained by log-rank test. [file crc-23-0167-s02.pdf]

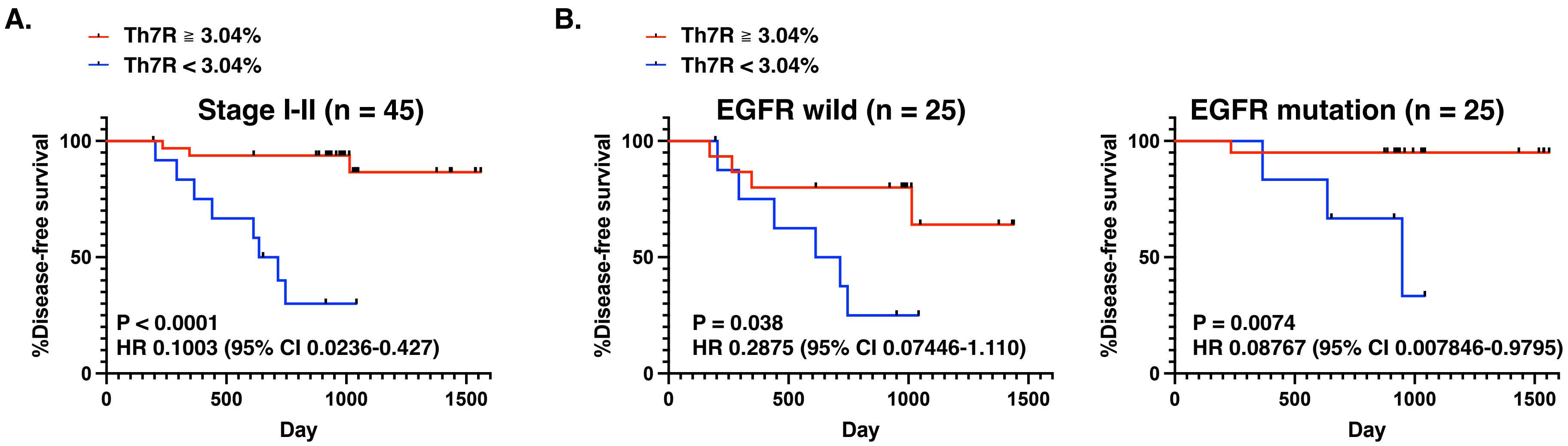

Fig. S2.

Kaplan-Meier analysis of disease-free survival (DFS) in patients with stage I-II disease (**A**), epidermal growth factor receptor (EGFR) wild-type disease, and EGFR mutations (**B**) using the threshold of preoperative %Th7R in peripheral blood obtained by receiver operating characteristic curve analysis. Significance was tested using the log-rank (Mantel-Cox) test. Hazard ratio (HR) and 95% confidence interval (CI) were obtained by log-rank test.
