# Supplementary material for: A Th1-like CD4+ T-cell Cluster That Predicts Disease-free Survival in Early-stage Lung Cancer
Source: Cancer Res Commun. 2023 Jul 19;3(7):1277–85. doi: 10.1158/2767-9764.CRC-23-0167 (PMC10355164; doi:10.1158/2767-9764.CRC-23-0167)
Supplement: Supplementary Figure S3 — Fig. S3. A: CD8+ T cells in peripheral blood collected before surgery were divided into four clusters according to CD62L and CD45RA expression and compared between no recurrence patients and recurrent patients: CD62LhighCD45RA+ naive, CD62LhighCD45RA- central memory (CM), CD62LlowCD45RA- effector memory (EM), and CD62LlowCD45RA+ effector memory with CD45RA expression (EMRA). B: Receiver operating characteristic curve (ROC) analysis was performed to determine if CD8+ T-cell clusters had the ability to discriminate between relapsed and non-relapsed patients. Then, Kaplan-Meier analysis of disease-free survival (DFS) was performed using the median value was used as a threshold because ROC analysis showed no significance. Significance was tested by log-rank (Mantel-Cox) test. Hazard ratio (HR) and 95% confidence interval (CI) were obtained by log-rank test. [file crc-23-0167-s03.pdf]

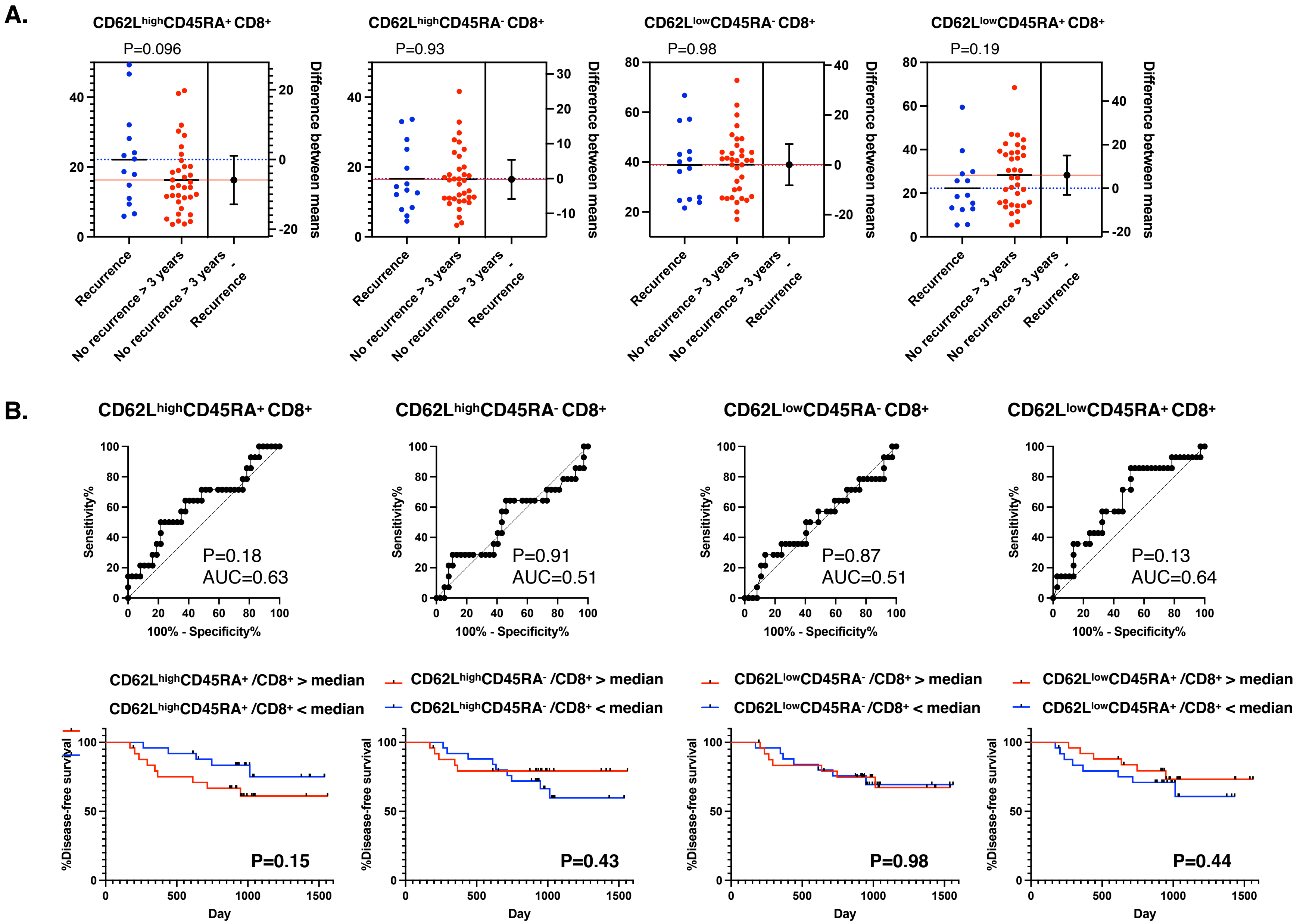

Fig. S3.

A: $CD8^+$ T cells in peripheral blood collected before surgery were divided into four clusters according to CD62L and CD45RA expression and compared between no recurrence patients and recurrent patients: $CD62L^{high}CD45RA^+$ naive, $CD62L^{high}CD45RA^-$ central memory (CM), $CD62L^{low}CD45RA^-$ effector memory (EM), and $CD62L^{low}CD45RA^+$ effector memory with CD45RA expression (EMRA).

B: Receiver operating characteristic curve (ROC) analysis was performed to determine if $CD8^+$ T-cell clusters had the ability to discriminate between relapsed and non-relapsed patients. Then, Kaplan-Meier analysis of disease-free survival (DFS) was performed using the median value was used as a threshold because ROC analysis showed no significance. Significance was tested by log-rank (Mantel-Cox) test. Hazard ratio (HR) and 95% confidence interval (CI) were obtained by log-rank test.
