# Supplementary material for: A Th1-like CD4+ T-cell Cluster That Predicts Disease-free Survival in Early-stage Lung Cancer
Source: Cancer Res Commun. 2023 Jul 19;3(7):1277–85. doi: 10.1158/2767-9764.CRC-23-0167 (PMC10355164; doi:10.1158/2767-9764.CRC-23-0167)
Supplement: Supplementary Figure S4 — Fig. S4. The percentages of CD8+ T-cell clusters in the peripheral blood were compared before and after surgical resection in patients with no recurrence and patients with recurrence. Differences were tested by paired Welch's t-test [file crc-23-0167-s04.pdf]

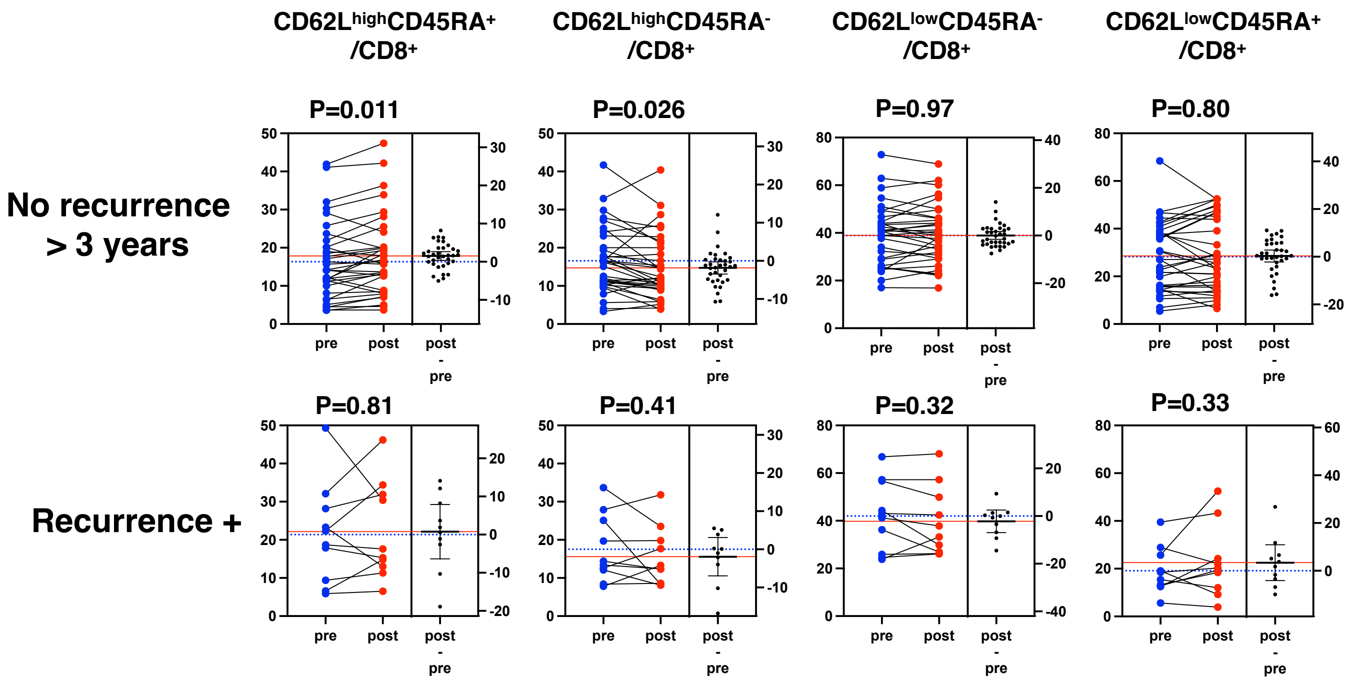

Fig. S4.
The percentages of CD8+ T-cell clusters in the peripheral blood were compared before and after surgical resection in patients with no recurrence and patients with recurrence. Differences were tested by paired Welch's t-test.
